# Blended learning in physical education: A systematic review

**DOI:** 10.3389/fpubh.2023.1073423

**Published:** 2023-03-09

**Authors:** Chen Wang, Roxana Dev Omar Dev, Kim Geok Soh, Nasnoor Juzaily Mohd Nasirudddin, Yubin Yuan, Xueyan Ji

**Affiliations:** Department of Sports Studies, Faculty of Educational Studies, Universiti Putra Malaysia, Selangor, Serdang, Malaysia

**Keywords:** blended learning, physical education, sports, educational technology, learning strategies

## Abstract

This review aims to provide a detailed overview of the current status and development trends of blended learning in physical education by reviewing journal articles from the Web of Science (WOS) database. Several dimensions of blended learning were observed, including research trends, participants, online learning tools, theoretical frameworks, evaluation methods, application domains, Research Topics, and challenges. Following the guidelines of the Preferred Reporting Items for Systematic Reviews and Meta-Analyses (PRISMA), a total of 22 journal articles were included in the current review. The findings of this review reveal that the number of blended learning articles in physical education has increased since 2018, proving that the incorporation of online learning tools into physical education courses has grown in popularity. From the reviewed journal articles, most attention is given to undergraduates, emphasizing that attention in the future should be placed on K-12 students, teachers, and educational institutions. The theoretical framework applied by journal articles is also limited to a few articles and the assessment method is relatively homogeneous, consisting mostly of questionnaires. This review also discovers the trends in blended learning in physical education as most of the studies focus on the topic centered on dynamic physical education. In terms of Research Topics, most journal articles focus on perceptions, learning outcomes, satisfaction, and motivation, which are preliminary aspects of blended learning research. Although the benefits of blended learning are evident, this review identifies five challenges of blended learning: instructional design challenges, technological literacy and competency challenges, self-regulation challenges, alienation and isolation challenges, and belief challenges. Finally, a number of recommendations for future research are presented.

## 1. Introduction

The integration of multiple technologies into traditional instruction has attracted enormous attention and offered numerous research avenues over the years. For instance, influential studies have confirmed the benefits of blended learning. According to Müller and Mildenberger (1), the definitions of blended learning most commonly used in scientific publications are those by Graham [(2), p. 5]: “blended learning is a combination of face-to-face and computer-mediated instruction” and by Garrison and Kanuka (3): “thoughtfully integrate the face-to-face learning experience in the classroom with the online learning experience.” Therefore, blended learning in this review includes technology-supported learning with the exception of fully online and fully face-to-face instruction. According to the sequence of integrating traditional classroom-based and online instruction, blended learning can be classified in the forms of blended, hybrid, flipped, or inverted. Despite the forms of blended learning, the use of blended learning has greater potential for transferring content into practice (4) and improves the quality and quantity of interaction between teachers and students (1), flexibility (5), learning engagement (6), and differentiated instruction (7) in classrooms.

To date, blended learning models are considered to be the most widely adopted instructional model by educational institutions as they are regarded as effective in providing flexible, timely, and continuous learning (8). The models have proven to be an upgrade from traditional learning models and fully online learning models as blended learning models combine the advantages of online and face-to-face learning (9). As a result, blended learning approach is referred to as the “new traditional model” or the “new normal” due to its advantages in optimizing the teaching and learning (10).

The significance of physical education in contemporary schooling is recognized internationally. Yang et al. (11) note that in addition to motor skills and physical fitness, physical education has a positive impact on students in several dimensions, such as their personal and social skills, patience, self-esteem, and self-confidence (12–14). In traditional teaching models of physical education, students are placed in a relatively passive position in order to receive knowledge and skills provided by the curriculum and the teaching content is inflexible as it ignores student differences and limits the opportunities for individual instruction and remediation by teachers (15, 16). To address the issue with the traditional teaching models of physical education, López-Fernández et al. (17) suggest blended learning models to provide students with personalized learning opportunities to optimize the quality of their learning in physical education classes, as well as to motivate students to learn.

A systematic review is necessary to understand current research situations of blended learning in physical education. Even though there have been considerable studies on blended learning in physical education, a systematic review of blended learning in this field is limited. To date, only one systematic review investigating the effectiveness of blended learning in higher physical education has been published (18). Therefore, this study aims to synthesize and analyze the findings to describe the current state and research trend of blended learning in physical education, and thus establish new directions for future research. This study was driven by the following research questions:

1. What are the research trends in blended learning in physical education?
2. Who are the main participants?
3. What are the main online learning tools?
4. What are the theoretical frameworks and evaluation methods used in blended learning in physical education?
5. What are the application domains and Research Topics involved in blended learning in physical education?
6. What are the reported challenges of blended learning in physical education?

## 2. Methodology

### 2.1. Search process

This systematic review follows the guidelines of the Preferred Reporting Items for Systematic Reviews and Meta-Analyses (PRISMA) (19). The search on the Web of Science (WoS) electronic database for the articles began in July 2022 and concluded in August 2022. WoS electronic database was chosen because of its high reputation and reliability in investigating leading articles. A search string was developed according to researchers' understanding and knowledge in the field of blended learning and physical education, as well as relevant blended learning and physical education search strings reported in other studies such as in Rasheed et al. (8) and Yang et al. (11). The search strings: (blended learning OR blended course OR hybrid learning OR hybrid course OR flipped learning OR flipped learning OR flipped classroom) AND (physical education OR sport^*^ OR physical activity^*^ OR exercise), were inserted in the advanced search query of the Web of Science database. The field option was then specified as a topic and restricted the search to the Social Sciences Citation Index. Then, the references of the papers included in this study were reviewed to ensure that the selected papers answered the six research questions of this review.

### 2.2. Eligibility criteria

To be considered for inclusion in this review, selected journal articles had to meet the following criteria: (a) define blended learning as the incorporation of traditional face-to-face and online learning, (b) related to blended learning in sports or physical education, (c) empirical study of SSCI indexing, and (d) published in English. On the other hand, the exclusion criteria included: (a) articles with sole concern on the face-to-face portion of blended learning, (b) book chapter reviews, meeting abstracts, reports, and review articles, (c) non-English articles, and (d) unavailable full-text articles.

### 2.3. Study selection

**Figure 1:**
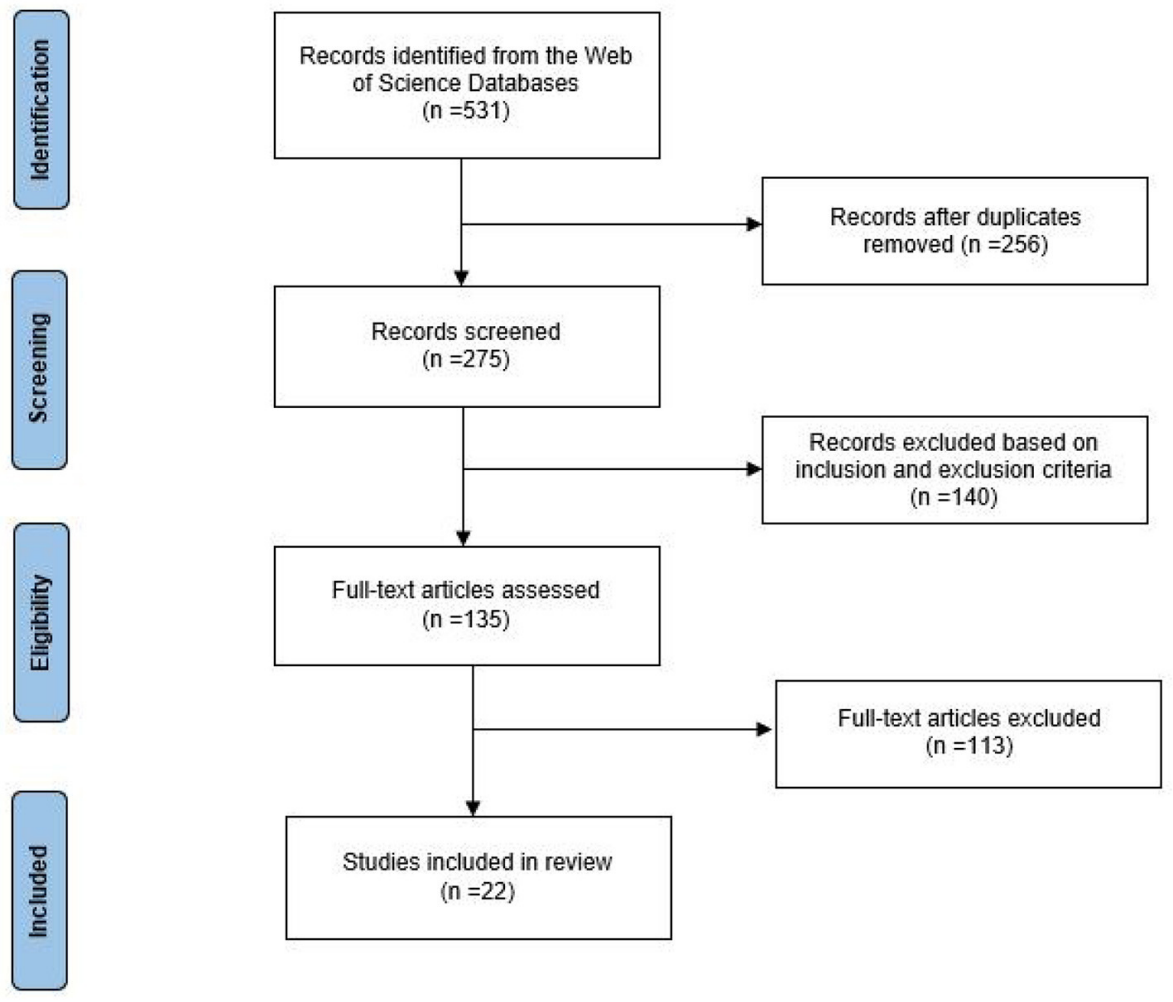
A review process based on the PRISMA statement.

A total of 531 journal articles were identified from the Web of Science database. A total of 256 duplicate articles were removed after considering the articles following the inclusion and exclusion criteria. Then, using the EndNote reference management software, a database of 135 articles with their titles, abstracts, and full text was created. The articles were carefully read and 22 articles were found pertinent to this review. Figure 1 shows the filtering process of this review based on the PRISMA statement (19).

### 2.4. Data extraction and quality assessment

The data extraction process included the identification of (a) the article's author, nationality, and publication year, (b) participants (i.e., K-12 students, undergraduates, teachers, and others), (c) online learning tools (i.e., learning platforms, learning software, recorded lectures, online learning materials, and others), (d) theoretical frameworks and evaluation methods (i.e., interviews, questionnaires, tests, and other methods), (e) application domains (i.e., basketball, football, badminton, and other courses) and Research Topics (i.e., perceptions, satisfaction, learning effects, and other items), and (f) challenges.

As the reviewed articles differed in research design, a quality assessment tool developed by Rowe et al. (20) that has been proven to be a useful tool for assessing qualitative, quantitative, and mixed methods was utilized (21). The tool assesses five important methodological aspects of a study, namely the background or literature review, sample, study design or methodology, outcome measures, and conclusions (see Table 1). The total score ranges from 0 to 5, with the higher scores representing better methodological quality. Articles scoring 4 or 5 are considered to be high in quality, articles scoring 3 are considered to be of moderate quality, and studies scoring between 0 and 2 are considered to be low in quality. In this review, two trained reviewers independently assessed the quality of the article, with disagreements resolved by the third reviewer. All 22 articles received a score between 4 and 5, indicating their high methodological quality.

**Table 1:** Methodological quality assessment tool.

**Criterion**	**Score**
1. Background/literature review:	
A. Detailed?	1
B. Limited?	0
2. Sample:	
A. Well-described?	1
B. Poorly described?	0
3. Study design or methodology:	
A. Clear?	1
B. Not clear?	0
4. Outcome measures:	
A. Valid/reliable and well-described?	1
B. Not valid/reliable, poorly described or not identified?	0
5. Conclusions:	
A. Supported by the study results?	1
B. Not supported by the study results?	0
**Methodological quality**		
**High**	**Moderate**	**Low**
Total score ≥ 4	Total score = 3	Total score ≤ 2

^*^Total score = sum of individual scores.

## 3. Results

This part reports the current state of blended learning in physical education and the key findings by addressing the six research questions of this review. The summary of the characteristics of the 22 studies involved is shown in Table 2.

**Table 2:** Characteristics of the studies examined in the preset review.

**References**	**Country**	**Participants**	**Sample size**	**Learning tools**	**Theoretical framework**	**Evaluation methods**	**Application domains**	**Research Topics**
Sidman et al. (22)	USA	Undergraduates	602	Online Lecture	Self-determination theory (SDT)	Questionnaire	Physical activity and wellness	Exercise motivation
Reddan et al. (23).	Australia	Undergraduates	35	Online learning materials	/	Test/open-ended questionnaire	Sports coaching	Learning effects/perception
Hinojo-Lucena et al. (24)	Spain	Undergraduates	131	Moodle learning platform	/	Test	Physical education	Learning effects/attendance
Otero-Saborido et al. (25)	Spain	Undergraduates	66	/	/	Open-ended questionnaire	Physical education	Design and validate self-assessment tool
Griffiths et al. (26)	UK	Undergraduates	147	Online learning	/	Questionnaire/ interview	Football	Perception/skills and qualifications/career development
Lin et al. (27)	China	Undergraduates	114	Wisdom Master Pro 2.0 learning platform	/	Test/questionnaire/interview	Dance	Learning effects/self-efficacy/perception/satisfaction
Chiang et al. (28)	China	Undergraduates	326	Basketball learning software	/	Test	Basketball	Learning effects
Hsia et al. (29)	China	Undergraduates	173	Wisdom Master Pro 2.0 learning platform	WSQ-based flipped learning model	Test/questionnaire /interview	Dance	Learning effects/learning motivation/self-efficacy/satisfaction/task load/perception
Hsia and Hwang (30)	China	Undergraduates	129	Evernote learning software	ARQI-based flipped learning model	Test/questionnaire /interview	Dance	Learning effects/self-efficacy/task load/perception
Koh et al. (31)	Singapore	Teachers	8	Online website	Self-determination theory (SDT)	Interview	Basketball	Perception
Lucena et al. (32)	Spain	K-12 students (primary and secondary)	119	Videos + learning software	/	Questionnaire	Physical education	Learning effects
Segura-Robles et al. (33)	Spain	K-12 students (secondary students)	64	/	/	Test	Physical education	Learning effects/psychological needs in exercise/sport motivation/satisfaction
Sargent and Casey (34)	UK	Teachers	2	Online materials and platforms	Constructivism theory	Interview/lesson observation/field notes/document analysis	Physical education	Perception
Yang et al. (35)	China	K-12 students (primary students)	80	Learning platform + robots	Hybrid learning theory	Test/questionnaire	Wushu	Learning effects /learning Interest/attitude
Lin et al. (36)	China	Undergraduates	75	Learning software	Cognitive apprenticeship and reflective practice theory	Test/questionnaire /interview	Billiards	Learning effects/motivation/self-efficacy
López-Fernández et al. (17)	Spain	Teachers	174	/	/	Questionnaire	Physical education	Perception
Calderón et al. (37).	Ireland	Teachers/undergraduates	123	Recorded lecture	Constructivism theory and post-humanism theory	Interview and reflective blog	Physical education theory (PET) curriculum	Perception
Chao et al. (38)	China	Undergraduates	290	TronClass learning platform	/	Test/questionnaire /interview	Dance	Learning effects, satisfaction and perception
Lin et al. (39)	China	Undergraduates	74	Learning platform	ICRA-based flipped learning model	Test/interview	Badminton	Learning effects and perception
Gallardo-Guerrero et al. (40)	Spain	Undergraduates	370	Online document	/	Questionnaire	Sports management	Interaction /perception
Finlay et al., (41)	UK	Undergraduates	203	/	/	Open-ended questionnaire	Physical education	Satisfaction and perception
Liu et al. (42)	China	Undergraduates	238	Superstar learning platform	3C model	Questionnaire	Physical Education Theory (PET) curriculum	Satisfaction

### 3.1. Research trends

**Figure 2:**
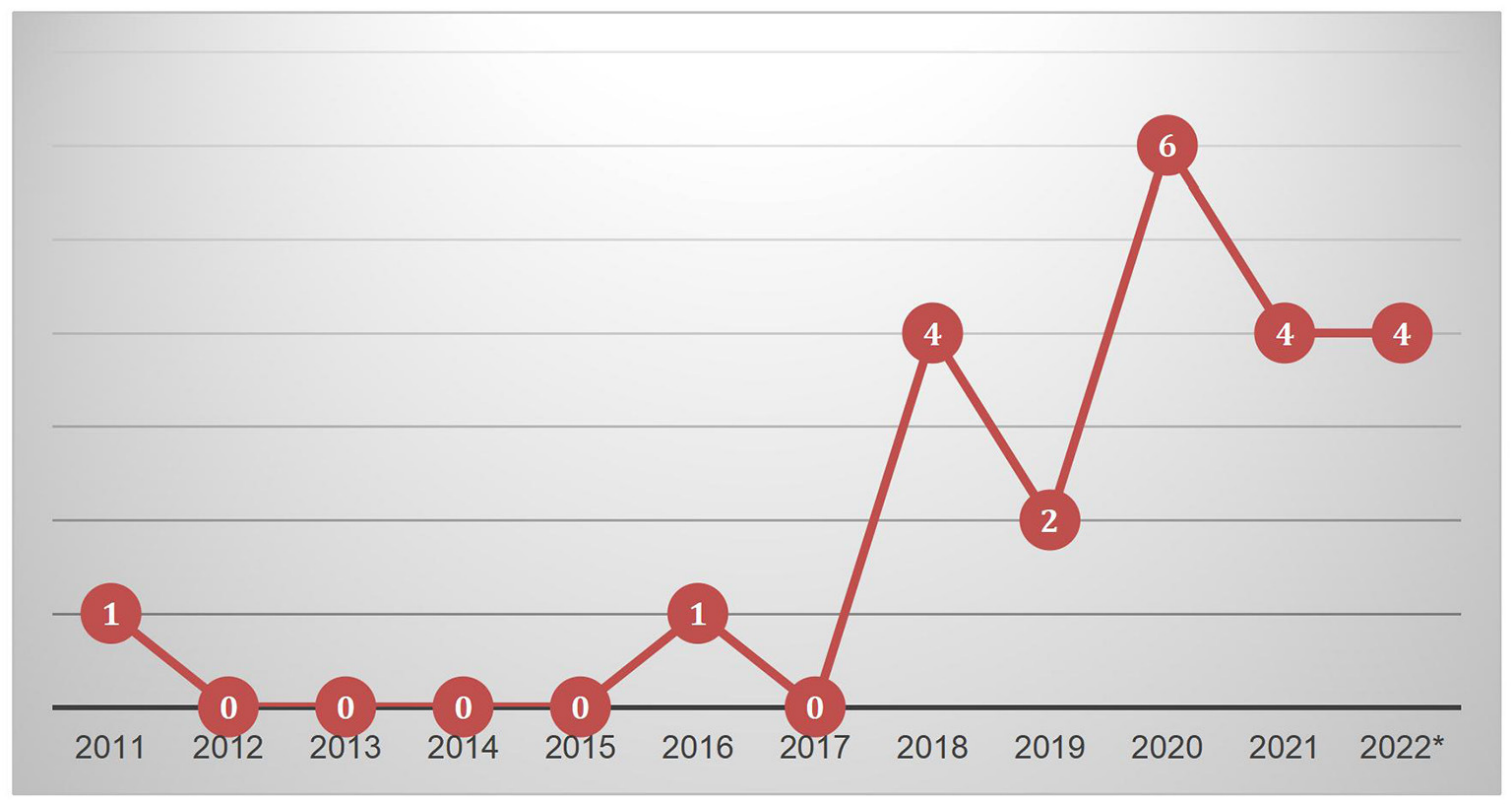
Number of articles published by year.

The first article on blended learning in physical education was published in 2011. However, since then, the research in this field was limited with zero publications in 2012, 2013, 2014, 2015, and 2017, and only one publication in 2016. However, beginning in 2018, physical education researchers have become increasingly interested in blended learning, with the number of articles reaching a peak in 2020. Journal articles published before August 2022 were also included. However, the number did not represent the accurate situation for the entire year of 2022 because this review concluded in August 2022. The graph of the trends in research on blended learning in physical education is shown in Figure 2.

Based on the number of publications on blended learning in physical education from 2011 to 2022, studies conducted in China accounted for 41 per cent of the total number of publications (*n* = 9). From the nine studies, Lin, Hsia, and Hwang authored five studies (27, 29, 30, 36, 39). The next highest number of publications on blended learning in physical education was conducted in Spain (*n* = 6) and the United Kingdom (*n* = 3), while each of the remaining studies was conducted in countries such as the United States, Singapore, Australia, and Ireland.

### 3.2. Participants

This review identified a total of 3,543 subjects enrolling in the 22 reviewed articles, with 2 (34) to 602 participants in each study (22). It is found that the majority of research subjects were undergraduate students (*n* = 15). A total of 5 articles reported detailed information about the majors of their participants and the locations of their degree programs, namely undergraduates of exercise science from Griffith University (23), undergraduates of physical education from the University of Granada, Organization of Educational Centers (Degree) (24), undergraduates of Pablo de Olavide University, Physical Activity and Sports Science (Degree) (25), undergraduates of sports management from San Antonio de Murcia University and Pablo de Olavide University (37), and undergraduates of sport and exercise science from the Edge Hill University (41). Out of the 22 reviewed articles, 3 articles focused on teachers, 1 article focused on teachers and undergraduates (37), and 3 articles focused on K-12 students. Among K-12 students, only primary and secondary students were included (32, 33, 35).

### 3.3. Learning tools

A variety of learning tools were used in the blended learning activities of physical education. Nine journal articles focused on learning platforms, such as Moodle, Wisdom Master Pro, TronClass, and Superstar as learning tools. Online learning materials, including online lectures, online documents, and online websites were studied in six articles. Learning software was mentioned in three articles, while one article used recorded lectures as the primary learning tool. Also, there were articles combining two learning tools (32, 34). The use of a learning platform and robots as learning tools was also studied in an article (35). Nevertheless, four articles did not report any learning tools.

### 3.4. Theoretical frameworks and evaluation methods

Blended learning is a pedagogical framework based on multiple theories of teaching and learning. This review discovered that the theories presented in the articles include self-determination theory (SDT) (22, 31), WSQ-based flipped learning model (29), ARQI-based flipped learning model (30), constructivism theory (34, 37), hybrid learning theory (35), post-humanism theory (37), cognitive apprenticeship and reflective practice theory (36), ICRA-based flipped learning model (39), and 3C model (42). However, of the 22 articles included in this review, 12 articles did not report a theoretical framework that was used to guide their research and teaching practice.

In terms of evaluation methods, 11 articles on blended learning in physical education used only 1 assessment method, 5 articles used 2 assessment methods, and 6 articles used 3 or more assessment methods. Questionnaires were employed by the greatest number of articles (*n* = 15), with 3 of them open-ended questionnaires (23, 25, 41). The evaluation methods were followed by tests (*n* = 11) and interviews (*n* = 10). Other evaluation methods such as lesson observation, field notes, document analysis (34), and reflective blogging (37) were also used.

### 3.5. Application domains and research topics

The range of applications for blended learning in physical education was diverse. There were 10 articles involving sports courses such as the Physical Activity and Wellness course (22), Sports Coaching course (23), and Sports Management course (40). There were also two articles on theory courses (37, 42). In addition, most of the current blended learning articles explored dancing (27, 29, 30, 38), followed by basketball (28, 31), football (26), Wushu (35), billiards (36), and badminton (39). A total of seven articles did not refer to specific areas of the physical education (17, 24, 25, 32–34, 41).

This review discovered that many articles investigated more than one Research Topic, and the totals exceeded the number of reviewed articles. As a result, the current review grouped the Research Topics of the 22 articles on blended learning in physical education into seven categories. The first category is the perceptions of students or teachers. This topic was investigated in 13 articles and was the most important concern of the blended learning community. The second category was the effects of blended learning in physical education on student learning. This topic was investigated in 12 articles. A total of 6 investigated the third category of blended learning in physical education which is student satisfaction with blended learning. In addition, 4 articles examined the student motivation (22, 29, 33, 36) and self-efficacy (27, 30, 36), while 2 articles studied task load. Other Research Topics such as attendance (24), self-assessment tools (25), skills qualifications and career development (26), psychological needs (33), learning interest and attitude (35), and interaction (40) were also discovered.

### 3.6. The challenges of blended learning in physical education

This review identified five categories of challenges of blended learning in physical education. They were instructional design challenges, technological literacy and competency challenges, self-regulation challenges, alienation and isolation challenges, and belief challenges (see Table 3). First, instructional design challenges (*n* = 6) involved a set of challenges related to scientific planning and rationalization of all aspects of the teaching and learning process in advance, based on student learning characteristics and teacher teaching styles. The second category was technological literacy and competency challenges (*n* = 5), which relates to a range of challenges associated with student/teacher proficiency and competence in the appropriate use of technology for teaching and learning. The third category, self-regulation challenges (*n* = 2) involved a series of related student behaviors that prevent students from self-regulating the emotions, thoughts, and actions they plan to take in achieving their learning goals. Belief challenges (*n* = 2) included negative attitudes and perceptions of teachers or students about the use of technology for teaching or learning. Finally, alienation and isolation challenges (*n* = 1) involved a set of associated emotional discomforts suffered by teachers or students when teaching or learning outside of traditional classrooms, mainly caused by loneliness and isolation from others.

**Table 3:** The challenges of blended learning in physical education.

**Challenges**	**References**
Instructional design challenges	(23, 26, 29, 31, 34, 38)
Technological literacy and competency challenges	(17, 23, 29, 32, 42)
Self-regulation challenges	(29, 42)
Belief challenges	(17, 38)
Alienation and isolation challenges	(17)

## 4. Discussion

### 4.1. Summary of findings and discussion

In this systematic review of the adoption of blended learning in physical education, 22 journal articles retrieved from the Web of Science (WOS) database were analyzed and grouped according to research trends, participants, learning tools, theoretical framework, evaluation methods, application domains, Research Topics, and challenges. The publication trend shows that there has been a growing interest in blended learning in physical education since 2018. This indicates that researchers have recognized the role of technology in physical education and have sought to apply technology in physical education to meet student educational needs based on the current challenges and technological teaching resources offered by contemporary society (32). In addition, the paucity of high-quality literature suggests that research on blended learning in physical education is still in its infancy around the world. Of the 22 articles in this review, 9 were conducted in China, 6 in Spain, and 3 in the UK. Each of the other articles was published in countries such as the USA, Singapore, Australia, and Ireland. Also, previous research supports the view that studies on blended learning in skills-based subjects are very limited and somewhat disconnected (27, 31, 43).

For the participants, the majority of blended learning journal articles in physical education have focused on undergraduates. This is in line with the study by Yang et al. (11) which found that researchers were more concerned with mobile learning in higher physical education. However, only a limited number of articles investigated K−12 students and teachers separately. This review discovers that blended learning can be a challenge for K−12 students as they have poor self-control and are unfamiliar with the operation of online learning platforms, making it difficult for them to watch instructional videos independently before class. As a result, some articles report several suggestions for applying blended learning in the K-12 educational setting, including determining the duration of online learning based on student attention spans (44), designing simple and streamlined online courses to create organized learning environments that enable students to improve user experience and reduce cognitive load (45), connecting online learning content to student experiences (46), creating study groups in which the teacher sets a theme and the students participate in the learning in a group form to develop the awareness of active participation and the ability to collaborate (47), providing personalized support (48), and learning through games to develop skills and knowledge related to course objectives (49). One prominent suggestion by the reviewed articles is that applying blended learning allows for the facilitation of various types of interactions (50). Among them, student-student interaction refers to peer support and collaborative learning, student-teacher interaction consists of evaluation, motivation, guidance, and prompt feedback (51), student–online learning content interaction is the process of intellectual interaction with learning content, to promote students' learning (52), and student-interface interaction refers to the interaction between students and the technology used to deliver educational content (53).

In addition, there is a limited number of articles on blended learning in physical education focusing on teachers. This may be because the selection of teachers as subjects for the study is challenging for several reasons. For example, the sample size may be too small for quantitative analysis and some teachers may be reluctant to embrace new teaching models. Nevertheless, technologies, through blended learning, offer many new opportunities for teaching. Besides that the use of blended learning could improve teachers' attitudes toward the application of technology, and it could also enhance their ability to apply technology to physical education, which is crucial for their professional development (54). Therefore, future blended learning papers in physical education should place greater emphasis on the teacher community.

Blended learning as an innovative pedagogical model requires the application of emerging methods in practice to meet specific pedagogical requirements (55). This review observed that teachers use different teaching platforms and online learning resources when incorporating blended learning in physical education in order to meet their pedagogical goals. The frequency of “learning platform” ranked highest among the selected studies, followed by “online learning materials” and “learning software.” With the development of educational technology, many student-centered learning platforms (e.g., Moodle, Superstar) are adopted by teachers in different educational institutions. These learning platforms are supported by teachers because they are powerful, easy to use, and can meet the common needs of both teachers and students (56). In addition, online learning materials which include online lectures, online documents, and online websites have also become teachers' choices. Compared to online learning platforms, online learning materials are richer in content and more diverse in learning formats. Teachers can select appropriate materials according to their student learning interests and practical needs (57). Self-developed learning tools or learning materials appropriate for the delivery of the courses are also created by teachers. One article developed and applied a robot (35), one article used recorded lectures (37), and a total of three articles used instructional software (e.g., basketball teaching mobile application) as the primary learning tool for learning activities (28, 32, 36). In general, while research on blended learning in physical education prior to 2020 on learning tools was homogeneous, the form diversifies as teachers begin combining two learning tools to produce better learning outcomes beginning in 2020, with the increased number of blended learning studies in physical education.

The theoretical framework is an essential component of disciplinary inquiry as it provides researchers with a strong argument for the significance of a particular research question and guides the analysis and interpretation of the data collected (58). The variety of theoretical frameworks found in reviewed articles indicates that blended learning in physical education is still in the stage of theoretical exploration, especially with twelve articles failing to specify a theoretical framework or a theoretical model used in the studies. The most commonly cited theories in this study are the self-determination theory (SDT) (22, 31) and the constructivist theory (34, 37). The self-determination theory asserts that individual development and progress are achieved through the satisfaction of three basic psychological needs: autonomy (self-identity and autonomy of choice), relatedness (being loved and interacting), and competence (being perceived as effective and capable). Meeting these three needs in a learning task will significantly enhance students' intrinsic motivation (14). This is because, in blended learning, students can determine their own learning time and pace based on their preferences (autonomy) and individual learning levels (competence). Blended learning also allows for collaborative learning that provides a highly interactive learning environment that meets student needs for relevance (relatedness). In short, many studies support the existing literature that blended learning environments have a positive impact on students' cognitive learning outcomes and “needs” for competence, autonomy, and relatedness (59, 60).

On the other hand, constructivism, upholding the constructivist theory, believes that students do not passively acquire knowledge, but actively construct new understanding and knowledge through personal experience and social discourse and combine new information with existing knowledge (61). Blended learning emerged to overcome the disadvantages of passive learning in traditional physical education learning models and enhance students' learning experiences and build problem-solving skills for further practice by optimizing the combination of various learning modes. Applying constructivist theory to a blended learning environment, therefore, increases student interaction, learning efficiency, and quality (62). Post-humanist theory seeks to provide a new epistemology that is non-anthropocentric and rejects dualism as a central (63). Guided by this theory, researchers have a better understanding of the significance of online and face-to-face instruction in blended learning. Also, according to post-humanist theory, when introducing blended learning in physical education, teachers need to design and use an integrated approach so that all instructional elements, as well as their components (e.g., online instructional materials and face-to-face activities), are interacting, thus enhancing the learning experience of students (37). This review also discovers another theory associated with metacognition that stresses helping students master and reflects on their current learning situations in blended learning in physical education so that they can improve their skill performance. It is cognitive apprenticeship. Cognitive apprenticeship is an instructional model proposed by American cognitive psychologists Collins, Brown, and Duguid in 1989 that emphasizes the importance of the process by which teachers transfer skills to students. The reflective practice focuses on students' reflection on their performance in an ongoing practice for personal development.

In traditional physical education learning models, students can only passively accept knowledge and skills in the classroom. To extend the learning time and space, a new approach involving virtual learning environments has been proposed, which is the Collaborative Cyber Community (3C) model (64). This model highlights the importance of interaction and collaboration in a virtual environment where students can gain motor skills and knowledge and teachers can develop the competencies to guide students in technology-related instruction. In addition, some theoretical frameworks based on the flipped learning model were also included in some of the reviewed articles, such as the watch, summary, and question (WSQ) flipped learning model, the annotation, reflection, questioning, and interflow (ARQI) flipped learning model, and the identification, communication, reflection, and analysis (ICRA) flipped learning model. The watch, summary, and question (WSQ) flipped learning model aims to guide students to mark key points and difficulties when watching instructional videos and summarize and ask questions during the before-class stage to promote students' understanding of the learning content (29). Even though students can focus on understanding the learning content through WSQ flipped learning model, there is a lack of practical experience and reflection on motor skills. In contrast, practice videos in the annotation, reflection, questioning, and interflow (ARQI) flipped learning model facilitate students' ability to observe their sports performance from a spectator's perspective and critically reflect on their motor skills and internal experiences, thus allowing them to improve their performance (30). Similarly, based on the educational theory of reflective practice, the Identification, Communication, Reflection, and Analysis (ICRA) flipped learning model was developed to improve the effectiveness of flipped sports learning and to create pedagogies that are more suitable for motor skill learning (39).

Evaluation for learning is a method used for instruction that provides feedback to students and teachers to promote learning and guide the next stage of action. Feedback includes informal feedback (e.g., immediate verbal comments on student performance or behavior) and formal feedback (e.g., written feedback given at the end of a test and recorded as evidence for use by the student and the organization). Evaluation to facilitate learning involves high-quality peer assessment of learning with each other and self-assessment, with the results used as a basis for deciding what will be learned in the future (65). In terms of evaluation methods, this review found half of the articles used formal feedback (tests), with questionnaires and interviews being the most common of the other feedback methods. Other evaluation methods such as lesson observation, field notes, document analysis (34), and reflective blogging (37) were also mentioned, indicating the diversity of assessment methods of blended learning in physical education research. In addition, it is worth noting that five articles in this review used two evaluation methods, while six articles used three or more evaluation methods. This is in line with the current research trend where mixed methods research is increasingly valued in social science research as it provides a better understanding of what blended learning entails and how it can support student learning in a variety of ways (66).

In terms of the application areas of blended learning in physical education, the dynamic domain was explored the most, indicating that at this stage, the research on blended learning in physical education is mainly focused on physical exercise, which is in line with the characteristics of physical education. Even though studies have been investigating blended learning in single sports, such as dance, basketball, football, and Wushu, the sports categories are limited and lack richness. Moreover, this review discovers that the physical education theory (PET) curriculum is currently a less studied (37, 42), probably because it is mainly conducted in higher education. However, it still has a vital role to play in the development of physical education. These two articles on the physical education theory (PET) curriculum only used interviews and questionnaires to investigate teachers' and students' experiences and satisfaction, so future research could use other research methods such as experimental and mixed methods to further investigate students' effectiveness and depth of perception. Furthermore, three articles explored both theoretical and pedagogical activity aspects of the physical education curriculum, such as the Physical Activity and Wellness (22), Sports Coaching (23), and Sports Management (40). The findings showed that there are different specificities to the use of blended learning, particularly the collaborative nature between students, experiential learning, the increased autonomy of students in their learning process, and the greater effect of critical thinking. Students receive more guidance and feedback from teachers in classroom activities, which is impossible to achieve with traditional teaching methods.

The findings from the dimension of the Research Topic reveal that perceptions (*n* = 13), as well as learning effects (*n* = 12) and satisfaction (*n* = 6), have been the main concerns of researchers when conducting blended learning studies, in addition to motivation (*n* = 4) and self-efficacy (*n* = 4). This is largely in line with the study by Chen et al. (67) which flipped the science classroom and found that the researchers were more concerned with the student's learning effects, as well as their perceptions and attitudes/motivation. This is justified because blended learning is a new approach for most teachers and hence, it is essential to examine the impact of a relatively new pedagogical model on students' academic performance and perceptions. However, from the review of 22 articles, blended learning in physical education has generally met researchers' expectations. For instance, several studies mentioned the positive impacts of blended learning on students' learning effects, self-efficiency, interaction, and satisfaction (23, 27, 28, 32, 35), as well as their perceptions, motivation, and attitude (31, 36, 38, 41, 42). Furthermore, other topics such as the task load (29, 30), attendance (24), self-assessment tools (25), skills and career development (26), and psychological needs in sports (33) were also conducted. The findings show that blended learning in the field of physical education, though in a developmental stage, meets the expectations of researchers.

While the advantages of blended learning models in optimizing teaching and learning are evident in countless influential studies, incorporating technology into education also brings a degree of unease to students and teachers. The most common problem related to blended learning in physical education is the instructional design challenge. Researchers have recently begun to develop or use online technologies for teaching or training activities. However, due to its specificity and complexity, physical education is more difficult to design in blended learning than other academic learning activities (68). The research by Boelens et al. (69) identifies four key challenges in the design of blended learning environments: incorporating flexibility, facilitating interaction, facilitating the learning process for students, and creating an effective learning environment. The shortcomings of instructional designs such as a lack of variety in content (29, 34) and lengthy videos (23) are mentioned in several articles. Also, Liu et al. (42) report that students experience a sense of distance when involved in too many online learning activities. Tsai et al. (70) concur stating that online courses in blended learning should only be offered every 2 weeks so that students can learn on their own and, if they encounter problems, they can solve them through face-to-face interaction. Another challenge is the technological literacy and competency that have become necessary for teachers and students to pursue contemporary education. The findings of López-Fernández et al. (17), Lucena et al. (32), and Reddan et al. (23) emphasize the lack of literacy and competency among students and teachers in using technology. Liu et al. (42) mention that students are more conservative in enhancing their information-related skills, which affects their learning outcomes and satisfaction with the course. Similarly, Hsia et al. (29) highlight the need for blended-learning students to be technologically competent because incompetence with learning technology can be a barrier to students' success in blended learning.

Another challenge for students in blended learning is that they are expected to self-regulate their learning activities outside of face-to-face classes. Two articles specifically identified the types of self-regulation challenges, namely procrastination (42) and improper time management (29). It is worth noting that procrastination is considered a chronic habit of unnecessarily putting off things that need to be done (71). Students' procrastination behavior differs in traditional and blended models, as students in blended learning environments experience a more pronounced sense of transactional distance (8). Belief challenges in this study refer to the negative attitudes and perceptions of teachers or students regarding the use of technology for teaching and learning. As reported by Brown (72), the difficulties encountered in adopting technology may be seen as disruptive to teaching and learning. Teachers may think of blended learning as instruction that has two teaching sections to deal with. For example, some physical education teachers believe that blended learning meant extra work compared with traditional teaching (17). Chao et al. (38) also report that students are reluctant to accept pre-class preparation. Furthermore, past research has mentioned that student learning activities, such as homework and preparation before face-to-face lectures, are challenging due to the alienation and loneliness felt by students online. Similarly, the study by López-Fernández et al. (17) finds that alienation and loneliness were also a challenge for physical education teachers because they find it more challenging to establish social relationships, either between teachers and students or between students, in the blended learning model than in the traditional model. This view was confirmed by a previous study of blended learning in physical education, where teachers felt disconnected from students and expressed concerns associated with the potential lack of social relationships and learning opportunities for students in a virtual environment (73).

### 4.2. Limitation

First, this study is limited by the use of rich eligibility criteria and methodology to consider only high-impact journals. Referring to other databases such as Google Scholar, PubMed, or Scopus might have resulted slightly differently. Second, only articles written in English are chosen. Third, the definition of blended learning opted in this review is a combination of traditional and online learning, so articles that do not conform to this definition are excluded, such as those that only mention the face-to-face part of blended learning. Finally, the study only focuses on the application of blended learning in physical education, such as the development trends and the main findings of current research. Therefore, the results cannot be extended to all research dimensions. Nevertheless, this research should be adequate to provide a roadmap for future research on blended learning in physical education.

## 5. Conclusion and suggestions

According to the overall findings, blended learning is in the initial stages of its development in the field of physical education. This result can be seen in several ways. First, researchers around the world have tried to apply blended learning in physical education, but the number of high-quality studies is very limited. Second, the majority of participants in the studies of blended learning on physical education are undergraduates, and a limited number of studies have been conducted on other subjects such as K−12 students and teachers. This review also reveals that studies prefer to investigate proven learning tools and the materials chosen by teachers as pre-course learning materials based on their personal preferences. In terms of theoretical framework, half of the researchers in the field of blended learning in physical education tend to not mention any theoretical framework. In addition, many prefer adopting a single evaluation method, with questionnaires being the most common method. Moreover, the focus of most journal articles on blended learning in physical education are on the preliminary aspects of blended learning research, namely perceptions, learning outcomes, satisfaction, and motivation. This leaves room for further research. This review also discovers that the most studied item in most articles on blended learning in physical education is dance. However, the majority of studies take a broad approach by not mentioning any specific item of physical education. Finally, the most common challenges for students and teachers revealed in this review are instructional design challenges, technological literacy and competency challenges, self-regulation challenges, alienation and isolation challenges, and belief challenges. In conclusion, this review provides a foundation for the future development of blended learning models by demonstrating the current status and development trends of blended learning in physical education.

Based on the results and discussion of the current review, several recommendations regarding blended learning in physical education are presented. First, it is necessary to improve the skills and perceptions of teachers. It is also evident that the researchers are very concerned about student perceptions of blended learning and learning outcomes. Most teachers and students identify instructional design and technological literacy and competence as their most obvious challenges. This implies that teachers need more training to improve their course design and management of online classes, including the use of multiple technologies as instructional support tools and the design of learning activities with various strategies at different stages of blended learning. To further explore the impact of blended learning on physical education, future research needs to focus on other populations (K−12 students, teachers, and educational institutions) and situations in other countries or regions. Future research should also focus on the application of blended learning in static physical education. Furthermore, it is recommended that the potential of blended learning in other sports be explored. In terms of the Research Topic, apart from the perceptions and learning effects, other aspects such as psychological needs and influencing factors should also be investigated.

## Data availability statement

The original contributions presented in the study are included in the article/supplementary material, further inquiries can be directed to the corresponding author.

## Author contributions

Conceptualization, software, formal analysis, investigation, resources, and writing—original draft preparation: CW. Methodology: CW and YY. Validation, writing—review and editing, visualization, and project administration: CW and RO. Data curation: CW and XJ. Supervision: RO, KS, and NM. All authors have read and agreed to the published version of the manuscript.
